# Effect of Dalbavancin on Staphylococcal Biofilms When Administered Alone or in Combination With Biofilm-Detaching Compounds

**DOI:** 10.3389/fmicb.2020.00553

**Published:** 2020-04-17

**Authors:** Miglë Žiemytė, Juan C. Rodríguez-Díaz, María P. Ventero, Alex Mira, María D. Ferrer

**Affiliations:** ^1^Genomics and Health Department, FISABIO Foundation, Valencia, Spain; ^2^Servicio de Microbiología, Hospital General Universitario de Alicante, ISABIAL, Alicante, Spain; ^3^CIBER Epidemiología y Salud Pública, Madrid, Spain

**Keywords:** dalbavancin, *E*-test, biofilm, *Staphylococcus*, ficin, *N*-acetylcysteine, xCELLigence

## Abstract

Microorganisms grown in biofilms are more resistant to antimicrobial treatment and immune system attacks compared to their planktonic forms. In fact, infections caused by biofilm-forming *Staphylococcus aureus* and *Staphylococcus epidermidis* are a large threat for public health, including patients with medical devices. The aim of the current manuscript was to test the effect of dalbavancin, a recently developed lipoglycopeptide antibiotic, alone or in combination with compounds contributing to bacterial cell disaggregation, on staphylococcal biofilm formation and elimination. We used real-time impedance measurements in microtiter plates to study biofilm growth dynamics of *S. aureus* and *S. epidermidis* strains, in the absence or presence of dalbavancin, linezolid, vancomycin, cloxacillin, and rifampicin. Further experiments were undertaken to check whether biofilm-detaching compounds such as *N*-acetylcysteine (NAC) and ficin could enhance dalbavancin efficiency. Real-time dose–response experiments showed that dalbavancin is a highly effective antimicrobial, preventing staphylococcal biofilm formation at low concentrations. Minimum biofilm inhibitory concentrations were up to 22 higher compared to standard *E*-test values. Dalbavancin was the only antimicrobial that could halt new biofilm formation on established biofilms compared to the other four antibiotics. The addition of NAC decreased dalbavancin efficacy while the combination of dalbavancin with ficin was more efficient than antibiotic alone in preventing growth once the biofilm was established. Results were confirmed by classical biofilm quantification methods such as crystal violet (CV) staining and viable colony counting. Thus, our data support the use of dalbavancin as a promising antimicrobial to treat biofilm-related infections. Our data also highlight that synergistic and antagonistic effects between antibiotics and biofilm-detaching compounds should be carefully tested in order to achieve an efficient treatment that could prevent both biofilm formation and disruption.

## Introduction

Increased drug resistance of bacteria is significantly reducing the therapeutic efficacy of antibiotics (Stewart and Costerton, 2001). Commonly, this resistance is augmented in bacterial biofilms, which can be described as bacterial communities adhering to abiotic or biotic surfaces and encased in a self-produced extracellular matrix (Chung and Toh, 2014; Hall and Mah, 2017). This matrix is composed of polysaccharides, proteins, and extracellular DNAs and plays an important role in persistent chronic infections, resulting in serious health complications. In fact, bacterial cells embedded in a matrix are up to 1,000 times more resistant to antibacterial compounds compared to their planktonic form, leading to increased morbidity and mortality rates of various diseases, like those associated with implantable medical devices (Flemming and Wingender, 2010; Rodrigues, 2011).

A major cause of medical device-associated and chronic infections, resulting in both economical and clinical burden, is the biofilm formation capacity of *Staphylococcus aureus* and *Staphylococcus epidermidis* bacteria (Otto, 2013; Moormeier and Bayles, 2017). This capacity, in addition to the widespread dissemination of methicillin-resistant *S. aureus* (MRSA) and *S. epidermidis* (MRSE), emphasizes the necessity to investigate new antimicrobial compounds and combine different treatment strategies for increasing the therapeutic potential of conventional antibiotics (Bjarnsholt et al., 2013). For instance, several agents for cell detachment and breaking down of biofilm matrix have already been reported. Some of them cleave the essential components of the biofilm matrix, like polysaccharides, proteins, or extracellular DNAs, destroying its architecture (Kaplan, 2010; Fleming and Rumbaugh, 2017). Ficin, a non-specific fig tree plant protease, belongs to this group of anti-biofilm compounds and is able to disperse staphylococcal biofilms via enzymatic lysis (Baidamshina et al., 2017). Others employ microbial signals that disperse bacterial cells embedded inside the biofilm exopolymeric matrix, like nitric oxide in *Pseudomonas* biofilms or certain quorum sensing inhibitors (Brackman and Coenye, 2015; Zhu et al., 2019). Other anti-biofilm agents, like *N*-acetyl-L-cysteine (NAC), besides the ability to impair matrix architecture, have also antimicrobial properties against different pathogenic bacteria, making this molecule an interesting tool to confront biofilms (Dinicola et al., 2014; Blasi et al., 2016; Costa et al., 2017). In addition, a combination of biofilm-detaching compounds together with antibiotics could represent an alternative strategy for the effective treatment of biofilm-associated infections.

Dalbavancin is a new lipoglycopeptide class antibiotic used against many gram-positive pathogens including staphylococcal strains in clinical practice (Chen et al., 2007). It is also a long-action antibiotic that interferes with bacterial cell wall synthesis and does not require frequent administration, allowing weekly dosing and earlier patient discharge from the hospital (Seltzer et al., 2003). Although dalbavancin has been proposed as a promising agent in biofilm-mediated infections, susceptibility to this antibiotic has mainly been tested using traditional microbiological tests such as microdilution or agar-based tests. However, it is well established that bacteria behave differently in a planktonic state or when forming biofilms, and there is currently limited information on its efficacy on biofilm-embedded bacteria, with only a few studies available (Meeker et al., 2016; Knafl et al., 2017; Di Pilato et al., 2020), some of them in animal models (Darouiche and Mansouri, 2005; Baldoni et al., 2013). In some cases, the efficacy of dalbavancin or vancomycin has been shown to be low, with less than an order-of-magnitude decrease in viable counts of staphylococci (Kussmann et al., 2018). Thus, recent work has proposed the combination of antibiotics and biofilm-detaching compounds to treat biofilm-mediated infections (Chen et al., 2013; Roy et al., 2018), but this strategy has currently not been tested with dalbavancin. In addition, there is conflicting evidence about the comparative efficacy of dalbavancin and other antibiotics of common clinical use in staphylococcal infections, such as vancomycin (Darouiche and Mansouri, 2005; Kussmann et al., 2018).

Recently, we evaluated biofilm inhibition and induction in *S. aureus* and *S. epidermidis* strains using 10 conventional antibiotics and suggested that impedance-based real-time cell analysis (RTCA) could facilitate determination of antibiotic sensitivity when bacteria grow in biofilms, resulting in faster and more accurate assays and therefore more efficient antimicrobial therapy (Ferrer et al., 2017b). The aim of the current study was to describe the effectiveness of dalbavancin to prevent *in vitro* biofilm formation of staphylococcal strains (both sensitive and methicillin-resistant isolates) and compare its effect with other antibiotics that are frequently used in clinical practice against indwelling device-related infections. Our biofilm growth measurements were performed by impedance-based cell analysis and confirmed by more classical tests such as crystal violet (CV) staining and counting of colony-forming units (CFUs). In addition, the effect of two biofilm-disaggregating molecules, NAC and ficin, was tested in combination with the antibiotic, to evaluate the potential synergy of a combined therapy to treat staphylococcal biofilm infections.

## Materials and Methods

### Bacterial Strains and Growth Conditions

Supplementary Table S1 lists bacterial strains used for this study. Staphylococcal strains were grown on tryptic soy agar (TSA) plates and tryptic soy broth (TSB) at 37°C at 120 r.c.f. *S. epidermidis* strain 43040 was isolated at the Microbiology Department of the University of Elche (Spain), MRSA strains were isolated at the Microbiology Department of the Alicante General Hospital (Spain) from a catheter tip in patients diagnosed with indwelling device-related bacteremia. *S. aureus* CETC 240 (*S. aureus* ssp. *aureus* Rosenbach 1884) is a biofilm-positive strain isolated by FDA, which is methicillin susceptible, and a reference strain recommended to test antibiotic resistance.

### RTCA-Based Biofilm Analysis

Real-time biofilm analysis was performed using xCELLigence RTCA SP equipment (ACEA Biosciences) according to the manufacturer’s instructions. For biofilm formation assays, bacterial strains were grown overnight in TSB and diluted with filter-sterilized TSB supplemented with 0.25% of D-glucose (TSB-glu). The experiments were performed as previously described by Ferrer et al. (2017b). Impedance data were registered at 10-min time intervals for 20 h, and they were transformed into cell index (CI) values, which accurately correlate with biofilm mass (Ferrer et al., 2017a, b).

To evaluate antimicrobial efficiency on bacterial biofilms, five antibiotics with different mechanisms of action were tested: linezolid (Accordpharma), vancomycin (Pfizer), cloxacillin (Normon), rifampicin (Mavi), and dalbavancin (Angelini). One hundred microliters of each antibiotic diluted in TSB-glu (twofold dilutions to final concentrations from 32 to 0.0625 mg/L) was used as background for impedance measurements. Further, 100 μl of bacterial cell suspension (OD_600_ = 0.175) was added, reaching a final optical density of 0.0875. This optical density corresponds to 10^7^–10^8^ cells, depending on the strain. The lowest antibiotic concentration required to inhibit bacterial growth with a CI value ≤ 0.05 was considered as the minimum biofilm inhibitory concentration (MBIC) (Bjarnsholt et al., 2013; Ferrer et al., 2017b).

To test the antibiotic effect on already-formed bacterial biofilms, the experiments were performed as previously described (Ferrer et al., 2017a). Briefly, 100 μl of cell suspension (OD_600_ = 0.153) was used as background. Then, 75 μl of TSB-glu was added to each well, reaching a final OD_600_ = 0.0875, and biofilms were grown for 6 h (*S. aureus* 240), 7 h (*S. aureus* MRSA4), or 9 h (*S. epidermidis* 43040), corresponding to the exponential phase of biofilm growth of each strain at which time the antibiotics were added (25 μl of each dilution, reaching final concentrations from 32 to 0.0625 mg/L for each tested antibiotic). After the addition of antibiotics, CI was monitored for a further 20 h. Two replicates of each antibiotic concentration sample and two negative controls were included in each experiment.

### Antibiofilm Compounds Assays

For RTCA experiments, ficin (Sigma) at concentrations of 10, 100, and 1,000 mg/L and NAC (Sandoz) at final concentrations of 0.5, 1, 2, 4, and 8 g/L were used alone and in combination with dalbavancin (0.5, 4, and 32 mg/L). In short, ficin and NAC were diluted in TSB-glu to the corresponding concentrations, and 100 μl of each dilution was used as background when anti-biofilm substances were added at the beginning of the experiment. After the background was measured, 100 μl of cell suspensions (OD_600_ = 0.175) was added into the corresponding wells, and biofilm formation was monitored for 20 h.

When NAC and ficin were added at the exponential biofilm growth phase, 100 μl of corresponding cell suspensions was used as background as described above. After that, 75 μl of the TSB-glu was added, reaching a final OD_600_ of 0.0875. When bacterial biofilm growth reached an exponential growth phase, 25 μl of the different concentrations tested for NAC or ficin and their combinations with dalbavancin was added into the corresponding wells. After the addition of biofilm-detaching compounds, biofilm growth was registered for 20 h more. Two replicates of each condition and their respective controls were tested in each experiment.

The effect of NAC and ficin on planktonic bacterial growth was also measured, by means of an absorbance plate reader Infinite M200 (Tecan, Durham, NC, United States). Briefly, overnight bacterial cultures were diluted to OD_600_ = 0.175, and 100 μl of each cell suspension was added into the corresponding wells of 96-well plates. Then, 100 μl of biofilm-detaching substances was added to the final concentrations of 10, 100, and 1,000 mg/L for ficin and 0.5, 1, 2, 4, 8, 16, and 32 g/L for NAC. Ninety-six-well plates were incubated at 37°C with orbital shaking at 120 rpm, and bacterial planktonic growth dynamics were monitored for 20 h. Two replicates of each concentration were included as well as their respective controls.

### MIC Determination

To determine minimum inhibitory concentrations (MICs) of the tested antibiotics on *S. aureus* and *S. epidermidis* strains on solid media, the *E*-test (bioMerieux) method was used according to the manufacturer’s instructions, following Baldoni et al. (2013). Broth microdilution assays were performed in accordance with the European Committee on Antimicrobial Susceptibility Testing (EUCAST, 2018). MBIC (or BIC) was calculated following Bjarnsholt et al. (2013) and Ferrer et al. (2017b).

### Biofilm Quantification

In order to determine dalbavancin and ficin effect alone and in combination on preformed MRSA4 biofilms, 175 μl of bacterial suspension (OD_600_ = 0.0875) was inoculated in TSB-glu and grown in 96-well flat-bottom Ibidi ibiTreat μ-plates 89626 (Ibidi, Germany). These plates are coated with a thin polymer layer in order to assure better biofilm attachment. After 7 h of growth, 25 μl of dalbavancin, ficin, or the combination of these two compounds was added, reaching final concentrations of 32 mg/L and 1 g/L, and the biofilms were grown for an additional 24 h. After that, the supernatant was discarded, bacterial cells were washed using phosphate buffer saline (PBS, pH = 7.4) to remove unadhered cells, and bacterial biofilms were stained using 0.1% CV as previously described (Stepanovic et al., 2000). Biofilm mass was quantified by an absorbance plate reader, Infinite M200 (Tecan, Durham, NC, United States), at 610 nm.

### Viable Count Assay

To assess the number of viable unadhered, planktonic bacteria and biofilm-embedded bacteria, 175 μl of MRSA4 suspension (OD_600_ = 0.0875) was grown for 7 h in triplicate in the xCELLigence system. After that, 25 μl of ficin and dalbavancin at the corresponding concentrations was added as described above, and the biofilms were cultivated for an additional 24 h. The supernatant was then collected, and serial dilutions were prepared, using 100 μl of each dilution for plating onto TSA plates in triplicate.

To evaluate viable cell number in bacterial biofilms, biofilms were carefully rinsed using PBS buffer to eliminate non-adhered cells, resuspended with 200 μl of PBS and sonicated for 5 min in order to disrupt biofilm matrix, and the serial dilutions of each sample were plated on TSA plates in triplicate as described above and incubated at 37°C overnight. After that, CFUs were counted, averaged, and expressed as log_10_.

### Statistical Analysis

To study differences in the biofilm CI values, regression analysis was performed by a linear model, using the function lm (library stats) in the R statistical package (Calcagno, 2013) between 10 and 20 h of biofilm formation time. For biofilm inhibition/induction analyses of CFUs and CV staining, experiments were performed in triplicate with three independent repeats in each experiment. Statistical significance was assessed using Student’s *t*-test, where ^∗^*p* ≤ 0.0 and ^∗∗∗^*p* ≤ 0.001.

## Results

### Dalbavancin Effect on Staphylococcal Biofilm Formation

**FIGURE 1:**
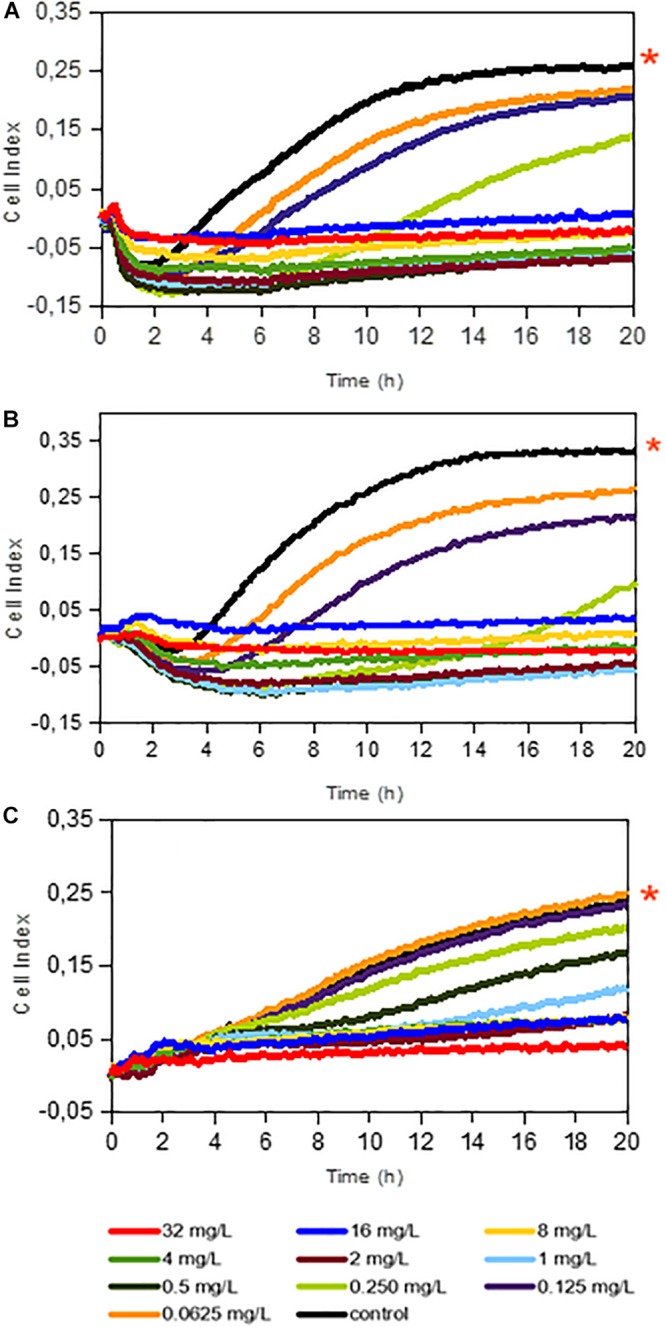
Dalbavancin effect on bacterial biofilm formation in *Staphylococcus aureus* 240 **(A)**, *S. aureus* MRSA 4 **(B)**, and *Staphylococcus epidermidis* 43040 **(C)** strains. The cell index (CI) values are measured by impedance in an xCELLigence equipment and correlate with total biofilm mass. Red asterisks indicate dalbavancin-free controls. Dalbavancin was added from the beginning of the experiment at concentrations from 0.0625 to 32 mg/L as indicated in the legend. Data are the means of two replicates.

Firstly, we evaluated dalbavancin’s effect on staphylococcal biofilm formation by real-time impedance analysis when the antibiotic was added together with the bacterial inoculum. Most of the tested *S. aureus* and S. *epidermidis* strains had similar biofilm growth dynamics with comparable CI (correspondent to biofilm mass) values. MRSA4 was the strain with the highest biofilm formation capacity (up to 26% higher CI than that of MRSA2 at 20 h), while the lowest CI values were observed for MRSA1 (Figure 1 and Supplementary Figure S1). The real-time dose–response experiments showed that dalbavancin is a highly effective antimicrobial and could prevent bacterial biofilm formation at low concentrations. The MBICs for the tested *S aureus* strains were between 0.5–1 and 2 mg/L for *S. epidermidis* strain 43040 (Figure 1 and Supplementary Figure S2). Table 1 shows the values of MBIC and MIC (as measured by *E*-tests) of dalbavancin for *S. epidermidis* and *S. aureus* strains, indicating that the MBIC is up to 22 times higher compared to the growth of the same strains on agar plates.

**TABLE 1:** Comparison between dalbavancin MBICs at 20 h of growth, as determined by impedance measurements, and MICs measured by standard *E*-test.

**Strain**	**MBIC (mg/L)**	**MIC (mg/L)**
*Staphylococcus epidermidis* 43040	2	0.094
*Staphylococcus aureus* 240	0.5	0.064
MRSA1	1	0.125
MRSA2	0.5	0.094
MRSA3	0.5	0.023
MRSA4	0.5	0.125
MRSA5	0.5	0.125

### Dalbavancin Effect on Biofilm Formation Compared to Other Antibiotics

**FIGURE 2:**
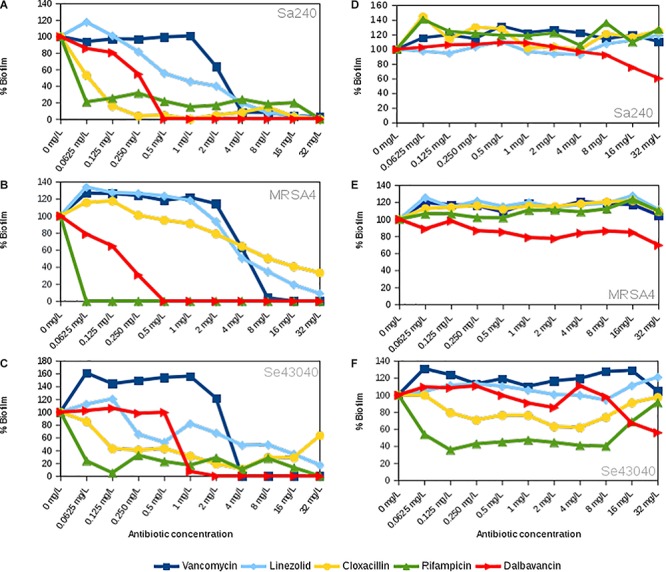
Concentration-dependent effect of linezolid, vancomycin, cloxacillin, rifampicin, and dalbavancin on biofilms in *Staphylococcus aureus* 240, *S. aureus* MRSA4, and *Staphylococcus epidermidis* 43040 strains. **(A–C)** The antibiotics were added at the beginning of the experiment together with the bacterial inoculum. **(D–F)** The antibiotics were added when biofilms were already formed, at their exponential growth phase. All charts indicate biofilm formation at 20 h of growth expressed as the percentage of cell index (CI) compared with the control without antibiotic. Values below 100% indicate biofilm inhibition, whereas values over 100% indicate biofilm induction, in comparison with the biofilm mass achieved in the absence of each antibiotic.

To further evaluate the dalbavancin effect on biofilm formation in *S. aureus* and *S. epidermidis* strains, we compared the effect of dalbavancin on biofilm formation to four antibiotics with different mechanisms of action, all of them commonly used in clinical practice: vancomycin, linezolid, cloxacillin, and rifampicin. For these experiments, we selected the MRSA4 isolate, which showed the highest CI values compared to the other MRSA strains (Supplementary Figure S2), together with methicillin-susceptible *S. aureus* strain 240 and the clinical isolate of *S. epidermidis* 43040. Figures 2A–C show the percentage of biofilm formation inhibition/induction relative to the antibiotic-free control for each strain (corresponding to 100% in the graphs). Although the antibiotic effect appeared to be strain specific, dalbavancin and rifampicin prevented biofilm formation in a dose-dependent manner, showing higher biofilm inhibition rates than vancomycin, linezolid, and cloxacillin. Cloxacillin was only effective against *S. aureus* strain 240 and could partially inhibit biofilm formation at some of the tested concentrations in *S. epidermidis* 43040. However, none of the tested concentrations of this antibiotic could eliminate preformed biofilm completely in this strain or in MRSA4. In fact, the exposure of MRSA4 to low concentrations of cloxacillin (0.06–0.125 mg/L) was not only ineffective but in fact also promoted biofilm formation up to 20% compared to the untreated control (Figure 2B). Additionally, neither linezolid nor vancomycin appeared to be effective against *S. aureus* and *S. epidermidis* biofilm development at low concentrations, and both tested antibiotics induced biofilm growth at concentrations <4 mg/L in strain MRSA4. It is important to underline that the MBIC for all tested antibiotics is considerably higher than that estimated by the traditional *E*-test method (Supplementary Table S2).

### Dalbavancin Effect on Established Biofilms

**FIGURE 3:**
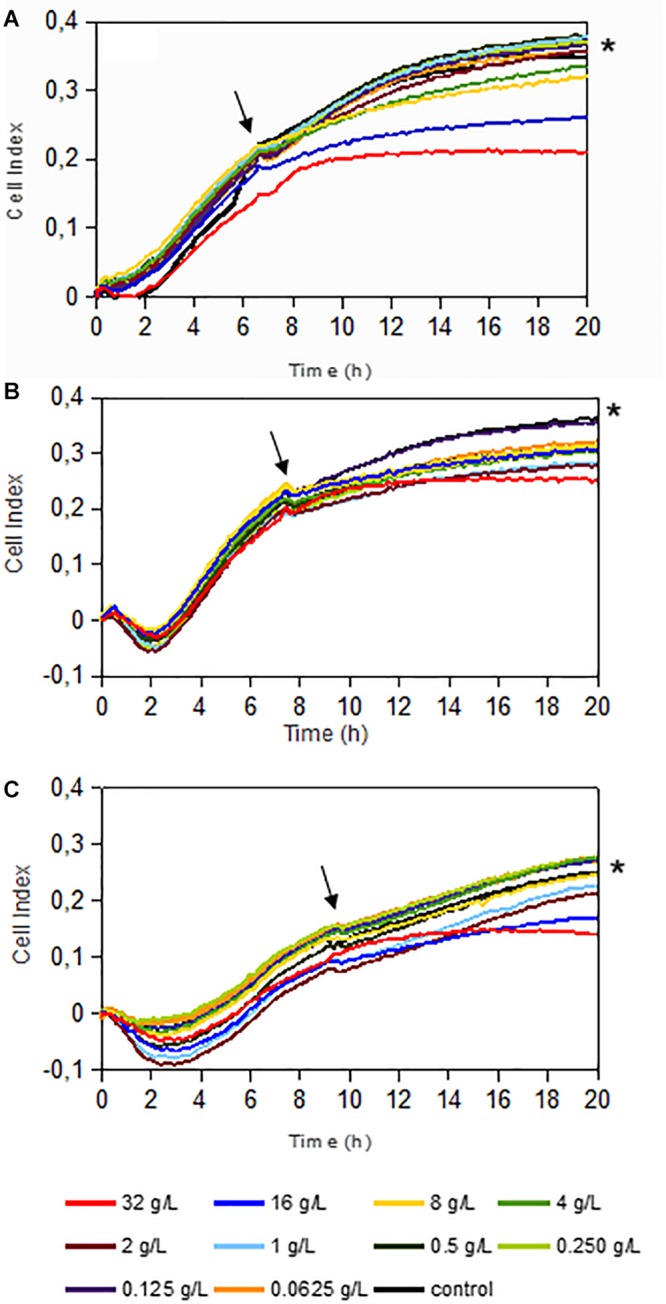
Dalbavancin effect on established staphylococcal biofilms in *Staphylococcus aureus* 240 **(A)**, *S. aureus* MRSA4 **(B)**, and *Staphylococcus epidermidis* 43040 **(C)** strains. Dalbavancin was added at the exponential growth phase of the biofilm (marked by black arrows) at 6 h (*S. aureus* 240), 7 h (*S. aureus* MRSA4), or 9 h (*S. epidermidis* 43040) at the concentrations shown in the legend. Black asterisks indicate the antibiotic-free cell control. Data are the means of two replicates.

It is known that some antibiotics have a limited efficacy to penetrate in established bacterial biofilms (Jefferson et al., 2005). For this reason, we further tested the effect of dalbavancin on already-formed staphylococcal biofilms. In our experimental setting, we considered established biofilms those which were on exponential biofilm growth phase (Ferrer et al., 2017a), that is, between 6 and 9 h depending on the strain, as previous work has shown that an exopolymeric matrix is fully formed by that time (Ferrer et al., 2017b; Gutiérrez et al., 2017). Figure 3 shows biofilm growth dynamics when different dalbavancin concentrations were added at the exponential biofilm growth phase. The data indicate that biofilm elimination was never achieved, but high concentrations of this antibiotic (8–32 mg/L) were able to reduce or fully prevent new biofilm formation and its further development in all the tested strains. Moreover, dalbavancin at 32 mg/L concentration was able to decrease the CI values over 40% compared to antibiotic-free controls after 20 h of inoculation (Figures 2D–F). The potential effect of dalbavancin was evident in the methicillin-resistant isolate MRSA4, where all tested concentrations resulted in biofilm growth reduction and partial elimination. Additionally, a strain-dependent effect was observed at low concentrations: whereas a concentration of 0.50 mg/L prevented new biofilm growth in *S. epidermidis* 43040, concentrations lower than 4 mg/L in *S. aureus* 240 turned out to be ineffective (Figures 2D–F, 3).

### Dalbavancin Effect on Established Biofilms Compared to Other Antibiotics

To evaluate the potential effect of dalbavancin in a comparative way, we performed dose–response experiments using conventional antibiotics on already-formed biofilms (Figures 2D–F). In contrast to dalbavancin, exposure of established MRSA4 biofilms to vancomycin, linezolid, cloxacillin, and rifampicin had no inhibitory effect at the maximum tested concentration of 32 mg/L. In addition, lower doses of these antibiotics (<32 mg/L) increased biofilm formation in this strain. The data indicate that dalbavancin was the only effective antimicrobial showing a strong biofilm inhibition capacity for this strain. Dalbavancin also halted new biofilm formation at 8–32 mg/L in *S. aureus* 240, while the other tested antibiotics resulted in increased biofilm growth at these concentrations. In the case of *S. epidermidis* strain 43040, both rifampicin and cloxacillin were able to decrease biofilm growth at the concentrations ≤8 mg/L (Figure 2F). Surprisingly, higher concentrations of these antibiotics (8–32 mg/L) had a limited effect in *S. epidermidis* 43040. The least effective antibiotic on preformed biofilm growth inhibition was vancomycin. This antibiotic induced biofilm formation of all tested strains (>30% relative to the antibiotic-free controls) at 20 h of biofilm growth.

### Combined Effect of Dalbavancin and Biofilm-Detaching Compounds

For this analysis, we selected an emerging therapeutic agent with mucolytic properties, NAC (Kundukad et al., 2017), and a natural plant protease, ficin, which has recently been described as an enzyme with unique properties to destroy the biofilm matrix (Baidamshina et al., 2017). Supplementary Figure S3 summarizes the effect of both anti-biofilm compounds on the biofilm formation of *S. aureus* 240, MRSA4, and *S. epidermidis* 43040 strains, when added at the moment of inoculation. Graphs show that all tested NAC concentrations induced biofilm formation in *S. epidermidis* 43040, while 8 g/L was able to slightly diminish biofilm formation in both Sa240 and MRSA4 strains. On the other hand, ficin (1,000, 100, or 10 mg/L) showed a notable effect on *S. aureus* biofilms, inhibiting their formation by 47% in Sa240 and 25% in MRSA4 strains, after 20 h of biofilm growth. On the contrary, this compound resulted in induction of *S. epidermidis* 43040 biofilm formation. Thus, the effect of this detaching compounds is species dependent.

**FIGURE 4:**
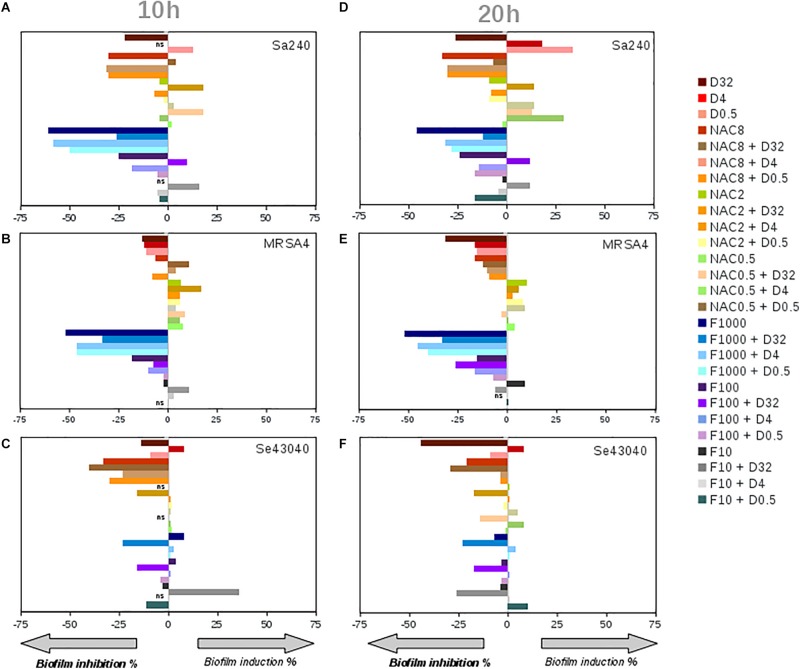
Effect on the biofilm growth of *Staphylococcus aureus* 240 **(A,D)**, *S. aureus* MRSA4 **(B,E)**, and *Staphylococcus epidermidis* strains **(C,F)**, respectively, of the biofilm-detaching compounds ficin **(F)** (1,000, 100, 10 mg/L) and *N*-acetyl-L-cysteine (NAC) (8, 2, 0.5 g/L), and the antibiotic dalbavancin **(D)** (32, 4, 0.5 mg/L) alone or in combination. **(A)**, **(B)** and **(C)** graphs correspond to the induction or inhibition values observed at 10 h of biofilm formation shown as percentage relative to the control, while **(D)**, **(E)** and **(F)** to 20 h of biofilm growth, respectively. On the *X* axis, zero corresponds to biofilm mass of antimicrobial-free controls at 10 and 20 h of biofilm growth on an xCELLigence 96-well plate.

Given that already-established biofilms are very difficult to eradicate, we next tested whether NAC or ficin alone or in combination with dalbavancin could have any effect on preformed staphylococcal biofilms. Figure 4 sums up the effect of dalbavancin and biofilm-detaching compounds separately and in combination when they were added at the exponential biofilm growth phase. Figures 4A–C represent CI values taken at 10 h of biofilm development, while Figures 4D–F represent those taken at 20 h. Dalbavancin alone at the concentration of 32 mg/L (represented as D32) greatly diminished new biofilm formation for all the tested strains. NAC, when administered alone on already-formed biofilms, also had an inhibitory effect on all three tested strains (Figure 4). However, when dalbavancin and NAC were combined, the inhibitory effect was dramatically hampered, and in *S. aureus*, it even led to biofilm induction. This suggests that the combination of NAC and dalbavancin is antagonistic.

**FIGURE 5:**
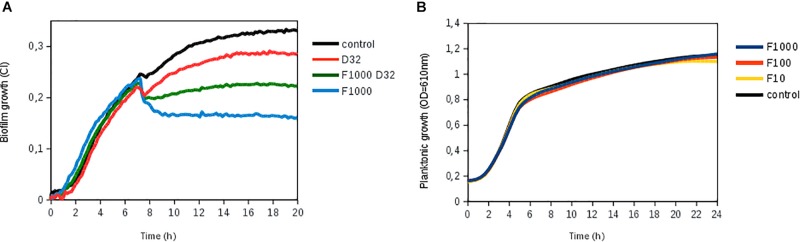
Biofilm disruption by ficin alone and in combination with dalbavancin. **(A)** Biofilm treatment by ficin [marked as F (1,000, 100, and 10 mg/L)] and dalbavancin [marked as D (32 mg/L)], either alone or in combination (F1000 D32), in methicillin-resistant *Staphylococcus aureus* strain MRSA4. Ficin, dalbavancin, or both was added on established biofilms after 7 h of growth in an xCELLigence equipment. **(B)** Effect of ficin on planktonic bacterial growth, measured as optical density in a 96-well plate. Bacteria were grown on TSB supplemented with 0.25% glucose at 37°C. Data are the means of three replicates.

**FIGURE 6:**
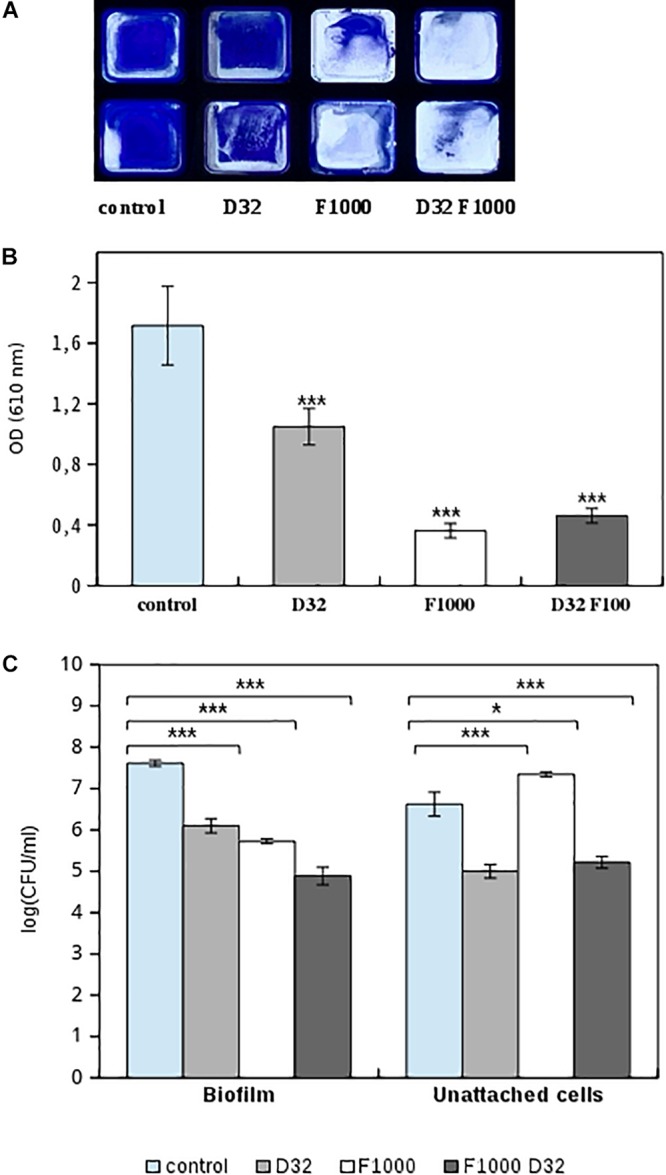
Effect of 1,000 mg/L ficin (marked as F1000) and 32 mg/L dalbavancin (marked as D32) on preformed MRSA4 biofilms as measured by CV staining and viable CFU counting. **(A)** MRSA4 biofilm quantification by CV in flat-bottom 96-well Ibidi plates, performed by duplicate. **(B)** Biofilm formation capacity after different treatments and CV staining (measured as optical density). Data show average values from three biological replicates. **(C)** Bacterial viability of biofilm and planktonic MRSA4 cells after treatment by ficin, dalbavancin, and their combination for 24 h. Data show the average of log CFUs from three replicates. Statistical significance was assessed by *t*-test; asterisks indicate *p ≤ 0.05 and ***p ≤ 0.001.

Ficin, when administered alone, had a significant effect in both *S. aureus* strains, preventing new biofilm formation up to 52% compared to an untreated control at both 10 and 20 h of biofilm growth (Figures 4A–E). However, ficin had no inhibitory effect on *S. epidermidis* 43040 biofilms. Interestingly, the combination of ficin with 32 mg/L of dalbavancin on this strain produces less inhibition than the antibiotic alone (Figure 4F), suggesting a potential counterproductive effect of both compounds. On the contrary, the combination of 32 mg/L dalbavancin and 1,000 mg/L ficin in *S. aureus* MRSA4 led to a significant improvement of biofilm inhibition relative to the antibiotic alone (*p* < 0.05) (Figure 5A), suggesting a potentiating effect. Although ficin alone produced higher biofilm reduction (Figure 5A), the detached cells were viable as they are not affected by this molecule (Figure 5B). This is confirmed by an increase in planktonic cells after ficin administration (Figure 6C). Thus, when using ficin, an effective antibiotic is needed in order to inactivate bacterial cells which are detached as a result of the enzyme’s activity and to prevent further colonization.

### Effect of Biofilm-Detaching Compounds on Planktonic Bacterial Growth

To investigate if NAC and ficin only hold biofilm-detaching properties or also have a direct antimicrobial effect on bacterial cell growth, we further assessed the effect of both compounds on planktonic bacterial growth. After the exposure of *S. aureus* 240, MRSA4, and *S. epidermidis* 4340 to different NAC concentrations (0.5–32 g L), it was observed that 8 g/L reduced bacterial growth over 50%, indicating that this compound alone has a strong antimicrobial effect. Bacterial growth was fully eliminated when the NAC concentration reached 32 g/L (MIC for all tested strains) (data not shown). On the contrary, none of the tested ficin concentrations (1,000, 100, and 10 mg/L) affected planktonic bacterial growth, indicating that ficin has a proteolytic effect only on the biofilm exopolymeric matrix, resulting in an efficient biofilm-embedded cell dispersal (Figure 5B).

### Comparison of Impedance Measurements With Classical Biofilm Quantification Methods

To verify that observed changes in CI are comparable to standard methodologies, we performed CV staining and CFUs of MRSA4 biofilms untreated or treated with ficin and dalbavancin alone and their combination. Figures 6A,B represent optical density measurements at 24 h of biofilm growth when ficin and dalbavancin were added. The addition of dalbavancin alone significantly decreased the number of adhered bacterial cells on 96-well plates. Reduced staining in the wells was also observed in cases where ficin was added alone and in combination with dalbavancin, confirming our previous observations of the ability of ficin to detach bacterial cells from biofilms. We also performed viable cell counting in both biofilm and unadhered bacterial cells in supernatants (Figure 6C). CFU counts showed that the viability of biofilm-embedded cells and planktonic cells was significantly affected by dalbavancin alone. When ficin was added alone, cell viability was not affected and a lower number of bacterial cells were observed in biofilms, together with a higher number of planktonic cells compared to the untreated control. These observations confirm the lack of antimicrobial properties of ficin and its biofilm-disaggregating activity. On the other hand, when ficin was added together with dalbavancin, the viability of both biofilm and unadhered cells decreased almost three orders of magnitude in biofilm cells and two orders of magnitude in unattached cells, showing a potentiating effect and suggesting that ficin increases the susceptibility of biofilms to this antibiotic.

## Discussion

Biofilm-forming capacity of staphylococcal strains contributes enormously to the pathogenesis of implant-associated infections, protecting these opportunistic pathogens from both immune system attack and antibiotic treatment (Gries and Kielian, 2017). Dalbavancin has already been described as an antibiotic with a potent *in vitro* bactericidal activity against many gram-positive pathogens, including MRSA and MRSE, in a planktonic mode of growth (Chen et al., 2007). However, the effect of dalbavancin on bacterial biofilms remains unclear as it has only been tested in a few occasions by using standard methods such as CV staining or MIC determinations (Fernández et al., 2016; Knafl et al., 2017). The impedance-based method performed in the current manuscript allows studying of the dynamics of biofilm formation and therefore the extent of biofilm reduction at different time points, obviating the need to select for a specific endpoint (Ferrer et al., 2017b). In this study, we evaluate the dalbavancin effect on the pattern and dynamics of *in vitro* biofilm growth in one *S. epidermidis* and six *S. aureus* strains and compare its efficacy to four different conventional antibiotics used in clinical practice (vancomycin, cloxacillin, linezolid, and rifampicin). Our experiments prove that dalbavancin and rifampicin were the best therapeutic agents against *S. aureus* and *S. epidermidis* biofilm formation in a dose-dependent manner when added at the beginning of biofilm growth. Interestingly, the superior efficacy of these two antibiotics is not related to their mechanisms of action, as rifampicin inhibits RNA polymerase (Campbell et al., 2001) and dalbavancin interferes with bacterial cell wall synthesis (Chen et al., 2007). Although rifampicin has been used in clinical practice against staphylococcal biofilm-related infections for almost three decades (Zimmerli and Sendi, 2019), this antibiotic should be administered carefully because of the danger of rapid emergence of rifampicin-resistant bacteria (Raad et al., 2007; Zhou et al., 2012). Thus, dalbavancin emerges as a promising solution in the fight against device-related infections, confirming the promising results obtained in animal models (Darouiche and Mansouri, 2005; Baldoni et al., 2013).

The impedance measurements performed in the current work show that, once the biofilm is formed, dalbavancin was the only tested antibiotic which could arrest new biofilm formation and prevent its further development in methicillin-resistant isolate MRSA4 and was effective in Sa240 and Se43040 at the concentrations of 8–32 mg/L. The other tested antimicrobials not only lacked an inhibitory effect on already-formed biofilms, but they also caused an induction of biofilm formation (Figures 2D–F). Given that some of these antibiotics have been shown to be able to penetrate through thick biofilms, their lack of inhibition could be due to a low metabolic activity of biofilm-embedded cells, which is known to decrease their susceptibility to antibiotics (Jefferson et al., 2005; Jacqueline and Caillon, 2014; Lopatkin et al., 2019). Thus, our data suggest that dalbavancin may act on established biofilms more efficiently than linezolid and vancomycin, which are among the most common antibiotics clinically administered for biofilm infections caused by *S. aureus* and *S. epidermidis* (Choo and Chambers, 2016). This may be facilitated by its mechanism of action, because this antibiotic not only inhibits bacterial cell wall synthesis but has also an ability to bind to bacterial membranes (Chen et al., 2013).

Given that even dalbavancin showed a limited efficacy to eradicate already-established biofilms, an effort was made to investigate its combined effect together with ficin and NAC, which have been reported to have biofilm-detaching properties. Unexpectedly, *in vitro* interactions between dalbavancin and biofilm-detaching compounds in preformed biofilms showed a dose and species-dependent effect (Figure 4). For example, the combination of NAC at concentrations of 8, 2, and 0.5 g/L with 32 mg/L of dalbavancin showed a decreased efficiency in inhibiting *S. epidermidis* 43040 biofilms compared to dalbavancin alone. This indicates that the combination of biofilm-detaching and antimicrobial compounds should be carefully tested in order to predict its combined effect.

We also observed that NAC had a direct antimicrobial effect on planktonic cells, while ficin did not inhibit bacterial growth at any of the tested concentrations (Figure 5B). This suggests that ficin is able to detach *S. aureus* biofilms by targeting only the biofilm matrix structure, in contrast to NAC. This should be considered when designing combined treatment strategies. For instance, our data demonstrate that ficin in combination with dalbavancin at final concentrations of 1,000 and 32 mg/L, respectively, showed an enhanced efficiency in the eradication of established MRSA4 biofilms compared to dalbavancin alone (Figure 5A). Although ficin alone provided an even greater biofilm reduction, its lack of antimicrobial activity implies that detached biofilm cells without the presence of an appropriate antibiotic could reach the bloodstream and result in serious medical complications. Therefore, we propose the combination of both a biofilm-detaching compound and an efficient antibiotic for maximal efficiency.

Altogether, the observations from the current manuscript show that dalbavancin has a strong activity against staphylococcal biofilms *in vitro*, making this antibiotic a promising agent to combat biofilm-mediated infections. Although its effect on already-formed biofilms is limited, ficin appears to intensify its efficacy, and the combination of dalbavancin with this or other disaggregating compounds should be further studied in the future. The differences obtained between agar-grown and biofilm-grown cultures underline that the use of appropriate biofilm susceptibility tests, such as those provided by impedance measurements, may offer a more accurate alternative for the selection of fast and individualized antibiotic treatment. Whether the use of impedance-based biofilm susceptibility tests allow earlier discharge from the hospital and lower rates of treatment failure should be clinically evaluated in the future.

## Data Availability Statement

The raw data supporting the conclusions of this article will be made available by the authors, without undue reservation, to any qualified researcher.

## Author Contributions

AM, JR-D, and MF conceived and designed the study. MŽ performed the experiments and analyzed the data. AM provided reagents. MV performed *E*-tests. MV and JR-D provided bacterial strains. MŽ, AM, and MF drafted the manuscript. All authors revised and approved the final manuscript.

## Conflict of Interest

The authors declare that the research was conducted in the absence of any commercial or financial relationships that could be construed as a potential conflict of interest.
