# Supplementary material for: Effect of Dalbavancin on Staphylococcal Biofilms When Administered Alone or in Combination With Biofilm-Detaching Compounds
Source: Front Microbiol. 2020 Apr 17;11:553. doi: 10.3389/fmicb.2020.00553 (PMC7180179; doi:10.3389/fmicb.2020.00553)
Supplement: Supplementary file 1 [file Data_Sheet_1.pdf]

Supplementary Table 1. Characteristics of the bacterial strains used in this study.

Strain	Species	Relevant properties	Source or reference
**43040**	*S. epidermidis*	Clinical isolate from catheter related bacteremia	Ferrer et al., 2016
**240**	*S. aureus*	Reference strain for antibiotic test	Human lesion isolated by FDA
**MRSA1**	*S. aureus*	Clinical isolate from catheter related bacteremia	This study
**MRSA2**	*S. aureus*	Clinical isolate from catheter related bacteremia	This study
**MRSA3**	*S. aureus*	Clinical isolate from catheter related bacteremia	This study
**MRSA4**	*S. aureus*	Clinical isolate from catheter related bacteremia	This study
**MRSA5**	*S. aureus*	Clinical isolate from catheter related bacteremia	This study

Supplementary Table 2. Minimum inhibitory concentration (MIC) values of vancomycin, linezolid, cloxacillin and rifampicin measured by the standard E-test protocols and expressed as mg/L. S and R in the table indicate if the strain was considered susceptible or resistant according to EUCAST guidelines.

	VANCOMYCIN	LINEZOLID	CLOXACILLIN	RIFAMPICIN
**SA240**	0.75 S	0.75 S	0.25 S	0.5 S
**MRSA4**	1 S	0.5 S	>2 R	0.006 S
**SE43030**	2S	0.19 S	>32 R	0.5 S

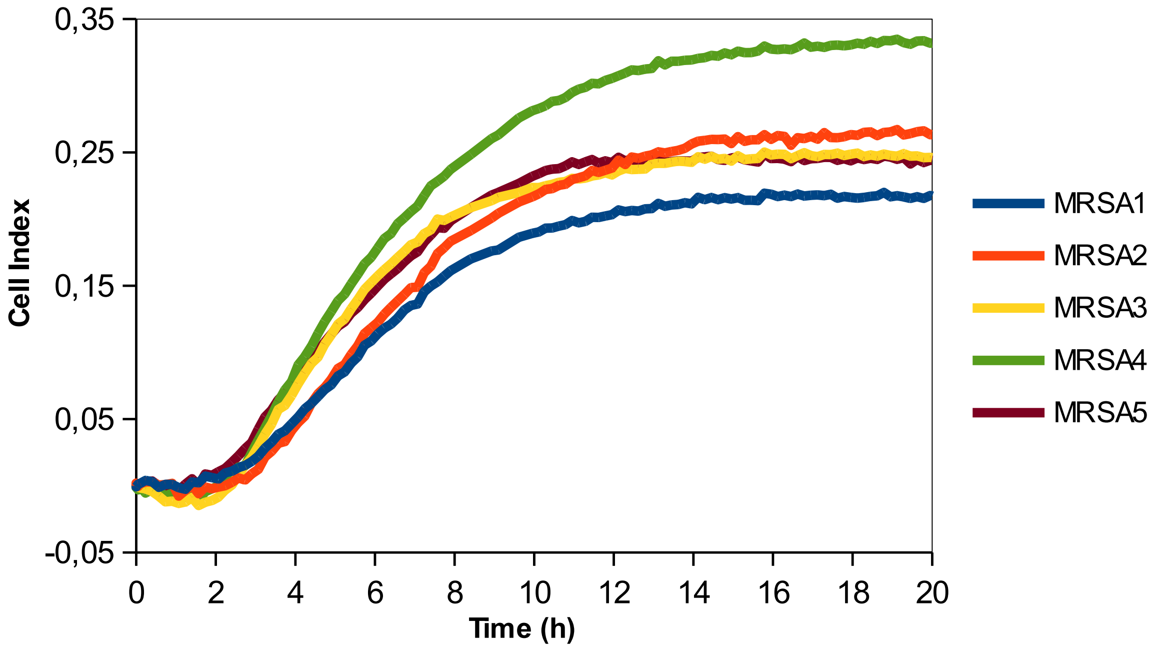

Supplementary Figure 1. Biofilm growth dynamics of methicillin resistant *S. aureus* strains (MRSA). Bacteria were grown on TSB supplemented with 0.25% glucose at 37°C. The Cell Index values are measured by impedance in an xCELLigence equipment and correlate with total biofilm mass. Data are the means of two replicates per strain.

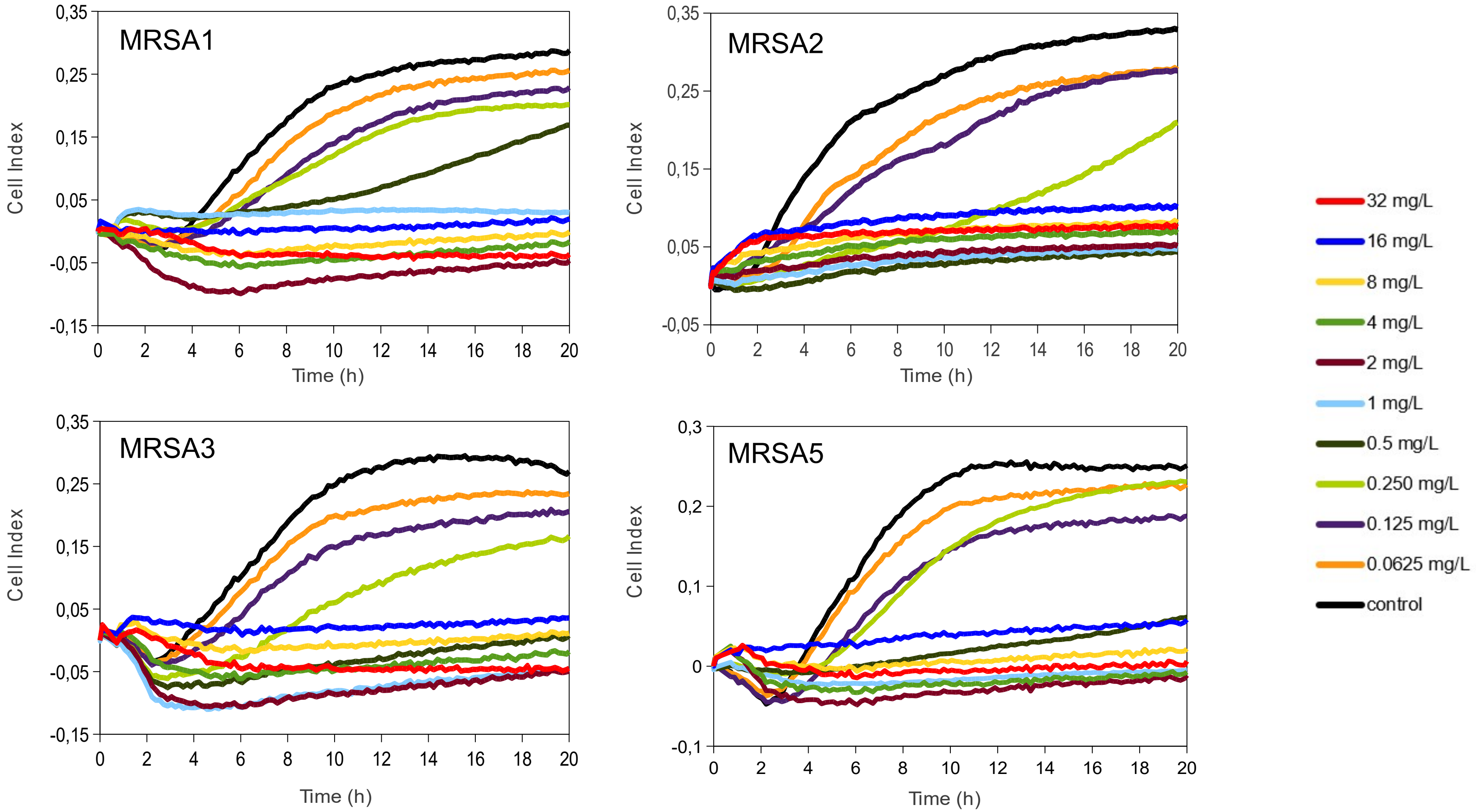

Supplementary Figure 2. Dalbavancin effect on bacterial biofilms in methicillin resistant *S. aureus* MRSA1, MRSA2, MRSA3 and MRSA5 strains. Dalbavancin was added from the beginning of the experiment at the concentrations indicated in the legend. Bacteria were grown on TSB supplemented with 0.25% glucose at 37°C. Data are the means of two replicates per strain.

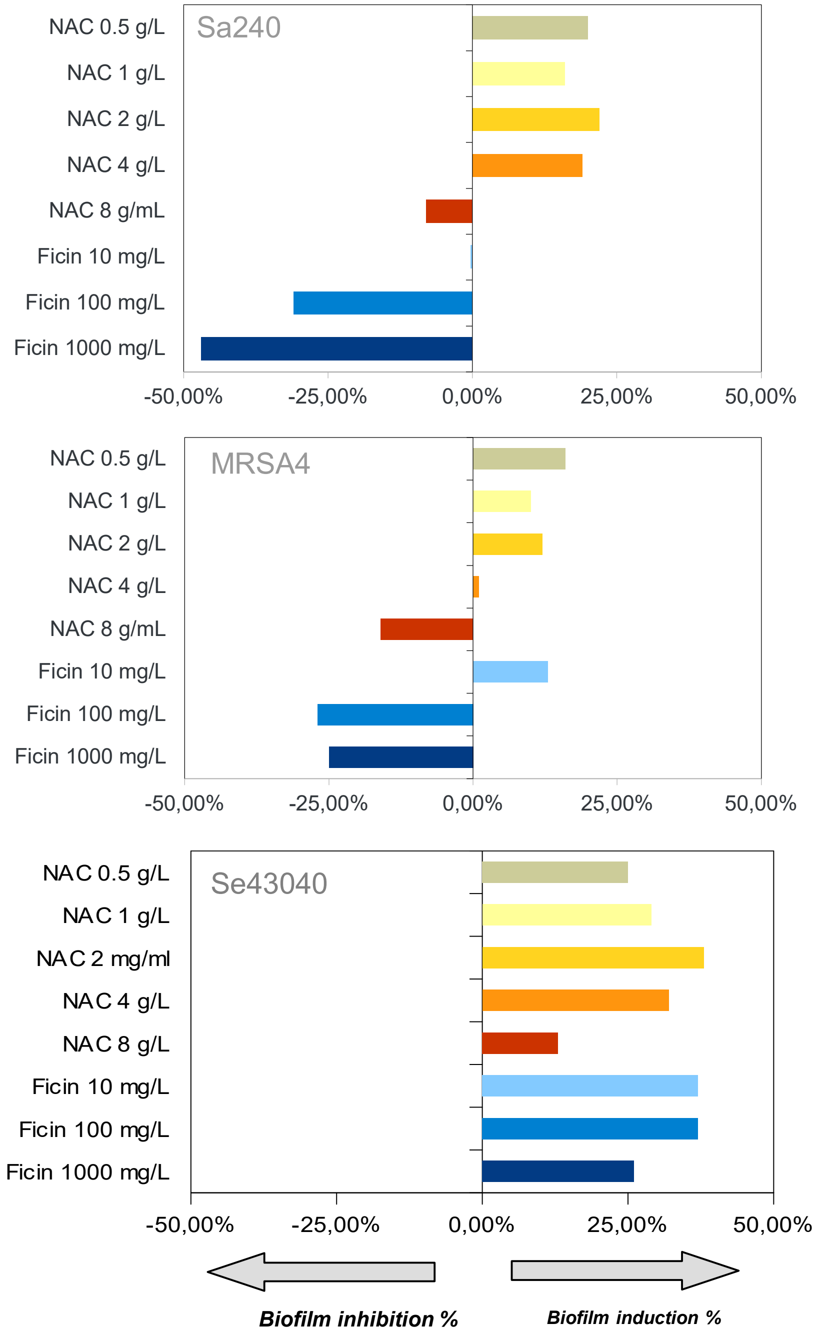

Supplementary Figure 3. Effect of anti-biofilm compounds NAC and ficin on biofilm growth of *S. aureus* strains Sa240 (A) and MRSA4 (B) and *S. epidermidis* 43040 (C) after 20h of biofilm growth. Biofilm detaching molecules were added at the beginning of the experiment at the concentrations indicated in the Y axis. Each bar represents the percentage of biofilm inhibition/induction compared to an untreated control. Biofilm growth was determined by impedance values in an xCELLigence equipment. All obtained values were statistically significant (n=5 per treatment, t-test, $p<0.05$).
